# Development of a Conductometric Sensor Based on Al,Ca-Doped ZnO for the Detection of Formaldehyde

**DOI:** 10.3390/s22197465

**Published:** 2022-10-01

**Authors:** Simona Crispi, Giovanni Neri

**Affiliations:** Department of Engineering, University of Messina, 98122 Messina, Italy

**Keywords:** zinc oxide, ZnO composite, formaldehyde, conductometric sensor, indoor quality air, workplace pollution

## Abstract

In the present study, the development of a conductometric gas sensor based on Al,Ca-doped zinc oxide composite which is finalized to the detection of formaldehyde (HCHO) at a low concentration in air is investigated. The electrical and sensing properties of the composite based on ZnO doped with different loadings of Al and/or Ca (from 0 up to 5 at%) were evaluated. The gas-sensing mechanism of Al,Ca-doped zinc oxide nanocomposite-based sensors was also discussed. The optimized 3%Al,3%Ca-ZnO sensor displayed a formaldehyde response of 3.5 (@ 4 ppm HCHO/air) and an experimental low detection limit of 125 ppb HCHO/air, at the operating temperature of 400 °C. The sensor was also shown to be selective to HCHO with respect to many interferent indoor gases, but NO_2_ changed the baseline resistance in an opposite way compared to the target gas. The developed device for monitoring HCHO in indoor and workplace environments has the advantage of a simple planar structure and can be easily fabricated for mass production by using low-cost materials and easy fabrication methods.

## 1. Introduction

Air pollution does not only affect the external environment that surrounds us, but those places that we consider safe. In fact, the air we breathe indoors such as in our homes, offices, and schools can be unhealthy. This specific pollution is called “indoor pollution” and can be quantified through the indoor air quality (IAQ) index. Attention to this issue has been highlighted by research on the increase in diseases related to the respiratory system in more sensitive subjects, such as children and the elderly, who spend more time indoors [1]. Formaldehyde is also widely used in numerous production processes, so it is a well-known occupational pollutant in the workplace [2]. 

Pollutants that worsen the indoor air quality generally come from chemical pollutants such as painted walls, furniture, and household cleaning detergents [2]. Among them, formaldehyde (HCHO) is one of the most toxic indoor and workplace pollutants. The International Agency for Research on Cancer (IARC) classifies formaldehyde as a carcinogen. In our homes, formaldehyde is present in very low concentrations, but it tends to accumulate mainly in conditions of low temperature and low humidity. The World Health Organization (WHO) guidelines for indoor air quality set a formaldehyde exposure limit (WHO, 2010) of 0.08 ppm (for an average concentration of 30 min). This limit is set so that there is no development of acute and chronic irritation of the respiratory tract, taking into consideration even the most sensitive subjects, including children and the elderly. In fact, concentrations of 0.4–1.0 ppm of formaldehyde could cause irritation to the eyes, nose, and throat. High concentrations, such as 6 ppm, could cause nasopharyngeal cancer, lung problems, and leukemia. In working places, HCHO has become one of the major threats for the worker’s health in the wood, textile, paper, construction, and chemical industries [2].

Many analytical methods have been developed for monitoring HCHO by using conventional analytical techniques, such as mass spectrometry and gas chromatography [3]. Optical techniques also show a low limit of detection (LOD) and a high accuracy and selectivity for the analysis of formaldehyde [4]. However, the cost and the high complexity and poor portability of these techniques are the main factors that limit their use. Therefore, simple and low-cost formaldehyde sensors should be mandatory. 

Conductometric sensors based on semiconductor metal oxides are particularly promising for detecting this pollutant and can ensure safe conditions at home and in the workplace. The operating principle is very simple and is based on the variations in resistance in the presence of gaseous species that act as electron donors or acceptors through surface reactions occurring on the surface of the semiconductor sensing material. Different gaseous species can be monitored with a single sensor by varying the operating temperature [5]. Furthermore, they have the advantage of being miniaturized devices and having low production costs. Among the metal oxides reported in the literature that can detect formaldehyde we can cite In_2_O_3_ [6], NiO [7], SnO_2_ [8], and ZnO [9], and so on. 

In this paper we focused our attention on the development of a reliable conductometric formaldehyde sensor for indoor applications based on doped-ZnO. The choice fell on ZnO as it is a simple material to obtain and has a low cost. It is an n-type semiconductor, has a 3.37 eV band gap, a 60 meV excitation binding energy, and a 400 cm^2^ V^−1^s^−1^ high electron mobility [10]. 

It is well known that ZnO is a sensing material with a wide application in conductometric gas sensors, due to its sensitivity towards several gases such as ethanol, nitrogen dioxide, acetone, and ammonia, etc. [11]. The addition of dopants modifies the electrical and gas-sensing properties of the zinc oxide [12]. In several papers in the literature, it has been shown that the presence of dopants belonging to the II and III groups of the periodic table improves the performance of ZnO due to the decrease of the grain boundaries in the lattice, therefore, improving the adsorption mechanisms of target gas. In a first exploratory study, Jaballah et. al., proposed a Ca-doped ZnO sensor for formaldehyde detection. The addition of this dopant has led to the improvement of sensing properties towards the target gas [9].

Here, we aimed to further improve the sensing performance of the zinc oxide for formaldehyde by adding different loadings of Al and Ca dopants. As these two dopants may introduce large changes in the electrical characteristics and interactions with different gases, the ternary Al,Ca-ZnO composite has been optimized previously by our group for monitoring other target gases, such as CO_2_ [13], resulting in an effective way to widen the sensing application of a pristine ZnO semiconductor. Focus was given to the optimization of the loading of Ca and Al and their ratio for increasing the sensitivity, decreasing the baseline resistance, and enhancing the selectivity to HCHO, compared to ethanol, the main pollutant interferent in the indoor environment. Furthermore, the study of the sensing mechanism and the reactions that occurred on the surface of pure and doped ZnO when dealing with formaldehyde, are also presented.

## 2. Materials and Methods

### 2.1. Synthesis of Al,Ca-Doped ZnO

The Al,Ca-doped ZnO composites were obtained by the sol-gel method following the synthetic procedure presented in the literature [10]. Briefly, 16 g of zinc acetate dehydrate [Zn(CH_3_COO)_2_·2H_2_O] was used as a precursor in 112 mL of methanol. For the doping aluminum nitrate-9-hydrate [Al(NO_3_)_3_·9H_2_O] and calcium chloride-6-hydrate [CaCl_2_·6H_2_O] were used in suitable quantities considering the atomic ratio percent (at%) of 1, 3, and 5 of [Al]/[Zn] and [Ca]/[ZnO] in 112 mL of methanol on magnetic stirring for 10 min. Subsequently, 200 mL of ethanol was added to the solution to be placed in an autoclave, maintained for 15 min under magnetic stirring, and dried in the supercritical conditions of ethyl alcohol. The obtained powders were then annealed in an oven for 2 h at 400 °C in air. The samples obtained were used for fabricating the conductometric sensors by depositing them on the interdigitated electrode platforms.

### 2.2. Characterization

Morphological analysis was carried out by SEM using a ZEISS 1540XB FE SEM instrument (Carl-Zeiss, Jena, Germany) equipped with an EDX detector. Transmission electron microscopy (TEM) was carried out with a JEOL JEM 2010 electron microscope (LaB_6_ electron gun) operating at 200 kV, equipped by a Gatan 794 Multi-Scan CCD camera for digital imaging. Samples for TEM analysis were prepared by dropping a suspension of the starting powder, dispersed in isopropanol and sonicated, on a 400 mesh holey-carbon coated copper grid. An X-ray diffractometry (XRD) analysis was carried out on a Bruker AXS D8 Advance diffractometer within the 2θ range of 20 to 80° using CuK_α_ as an X-ray source (λ = 1.5406 Å). ATR-FTIR analyses were performed using a Spectrum Two (Perkin-Elmer) FT-IR spectrometer with a diamond ATR (attenuated total reflection) single reflection accessory.

### 2.3. Sensing Tests

The sensing tests were carried out using a conductometric 3 × 6 mm ceramic platform with interdigitated contacts in Pt. Details on the conductometric ceramic platform have been reported in a previous paper [14]. Ten µL of nanomaterial dispersed in water was deposited on each device. To carry out the tests, the sensors were inserted in a holder connected to the instrumental apparatus. The instrumental apparatus consisted of a stainless-steel measuring chamber, a multimeter to acquire data Agilent 34970A, and a dual-channel power Agilent E3632A to bias the built-in heater of the device.

The sensors were subjected to a flow of dry air of 100 cc (20 cc O_2_ + 80 cc N_2_) in relation to the flow of formaldehyde gas adopted for the sensitivity tests. The formaldehyde gas bottle used for the pulse is certified by the SOL company (www.solgroup.com (accessed on 20 August 2022)). To validate our sensor, a commercial formaldehyde sensor (SFA30 electrochemical sensor from Sensirion AG, Switzerland) was used and located near the gas flow output of our sensor. 

The sensor response to target gas (R) is determined as the ratio of R_a_ (resistance of the gas sensor in dry synthetic air) to R_g_ (resistance of the injected gas sensor): R = R_a_/R_g_. The calculation of the response and recovery time, i.e., the variation of the sensor resistance over time until it reaches 90% equilibrium after the start and the end of CH_2_O detection, facilitated the study of the sensor dynamics.

## 3. Results

### 3.1. Morphological and Microstructural Characterization 

**Figure 1:**
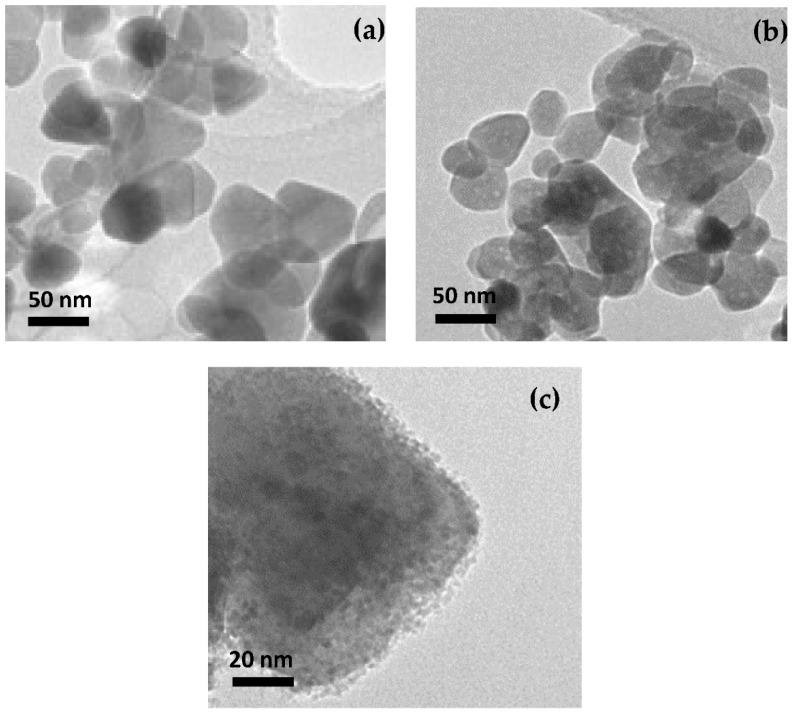
TEM images taken at the same magnification showing the particle shape and size of: (**a**) ZnO; (**b**) 1Ca-doped ZnO; and (**c**) Higher magnification of one particle of 3Al,3Ca-doped ZnO showing the fine particles dispersed on the surface of the bigger one (see text for the explanation).

The TEM images reported in Figure 1 highlight the differences between the pristine ZnO and the doped samples, in agreement with previous reports [10,11]. Well-shaped particles with a size range of 20–80 nm were observed on the ZnO and Ca-ZnO (see Figure 1a,b), whereas the presence of small particles of a few nm in size decorating the surface of the large ZnO grains was clearly noted on Al doped-ZnO and Al,Ca doped-ZnO samples (see Figure 1c) [10,15]. As these smaller particles were not found on the pure ZnO and Ca-ZnO samples, it is plausible to assume that they are related to the presence of phase(s) rich in Al, which are likely to come from a heterogeneous nucleation and successive growth on the surface of ZnO particles during synthesis in the presence of this dopant.

Of course, the presence of such segregated particles on the surface, as probed by TEM, means that the local/surface composition could be very different from the theoretical one. Indeed, the at% for each dopant was theoretically calculated and based on the ratio of precursor materials. Unfortunately, at this stage we did not have the support from XPS or other techniques, which could have given us important quantitative information. 

**Figure 2:**
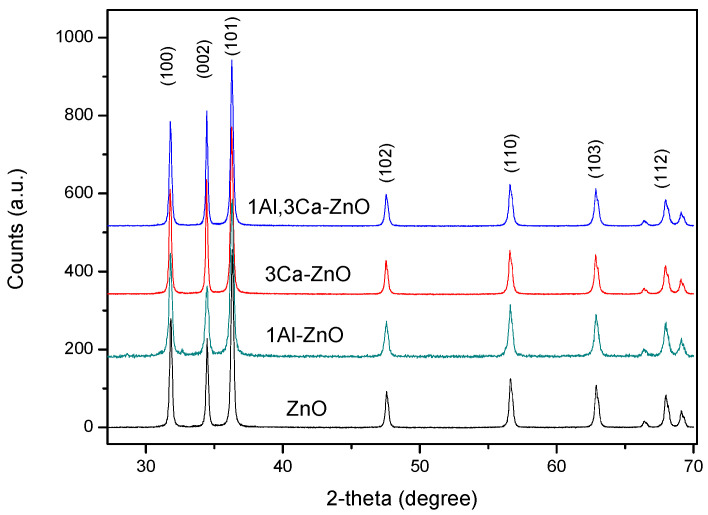
XRD of some representative samples used in this study.

The X-ray diffraction (XRD) spectra showed the polycrystalline nature of the doped-ZnO materials, which also maintained the wurtzite structure of pure ZnO (see Figure 2). A detailed XRD characterization of the samples investigated has been carried out and reported separately in various papers [9,13,15]. An effect of the doping was to reduce crystallite size. This finding has been reported by other authors, even when the Al,Ca-doped ZnO composites were prepared by different synthesis methods [12]. As an explanation of this, we may focus attention on the large difference in the atomic radius between zinc (1.42 Å) and calcium (1.94 Å), leading to lattice structure distortion, which consequently limits the growth of the wurtzite grains [16].

In summary, the morphological and microstructural variation occurring through the doping process are expected to give large benefits in gas detection due to an enhancement of the active surface sites. Further, the formation of contact zones at different conductivity modifies the local Schottky barrier at the grain boundaries, thus creating multiple pathways for electrical conduction and helping gas-sensing detection.

### 3.2. Chemical Characterization

**Figure 3:**
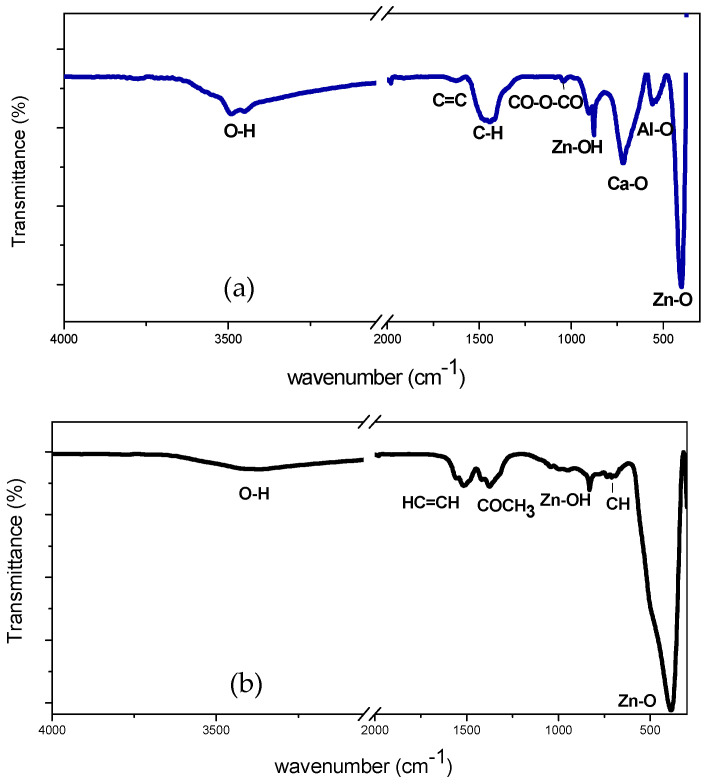
ATR-FTIR spectrum of: (**a**) ZnO; (**b**) 3Al,3Ca doped ZnO.

Additional information was acquired by a detailed ATR-FTIR characterization. In Figure 3a the IR spectrum of pure ZnO is reported, where the main peaks observed at 3398 cm^−1^ were relative to the O-H group, at 1513 cm^−1^ they were relative to the HC = CH group, and at 1372 cm^−1^ they were relative to the -COCH_3_ group [17]. In the fingerprint region the presence of additional peaks were found at 829 cm^−1^–700 cm^−1,^ relating to the Zn-OH, and at 400 cm^−1^, relating to the Zn-O [18].

Figure 3b shows the characteristic ATR-FTIR obtained from Al-Ca doped/ZnO samples. Peaks were observed at 3492–3442 cm^−1^ for stretching of the -OH group, at 1628 cm^−1^ of the C=C group, at 1440 cm^−1^ of the C-H group, at 1040 cm^−1^ of the CO-O-CO group, at 870 cm^−1^ of the Zn-OH group, at 719 cm^−1^ of the Ca-O group [19], at 555 cm^−1^ of the Al-O group [20], and stretching of the Zn-O group.

Bands associated with organic functional groups detected on the sensor layer before exposure to HCHO were due to some residues coming from the precursor materials used for the sol-gel synthesis, e.g., Zn(CH_3_COO)_2_ and methanol.

Although the ATR-FTIR spectra showed that it can only give a qualitative representation of the samples investigated, this technique could be utilized to characterize the composite materials under HCHO sensing working conditions and give some information about the adsorption of formaldehyde on the surface of the sensing layer (see in the paragraph below). 

### 3.3. Electrical and Gas Sensing Characterization

**Figure 4:**
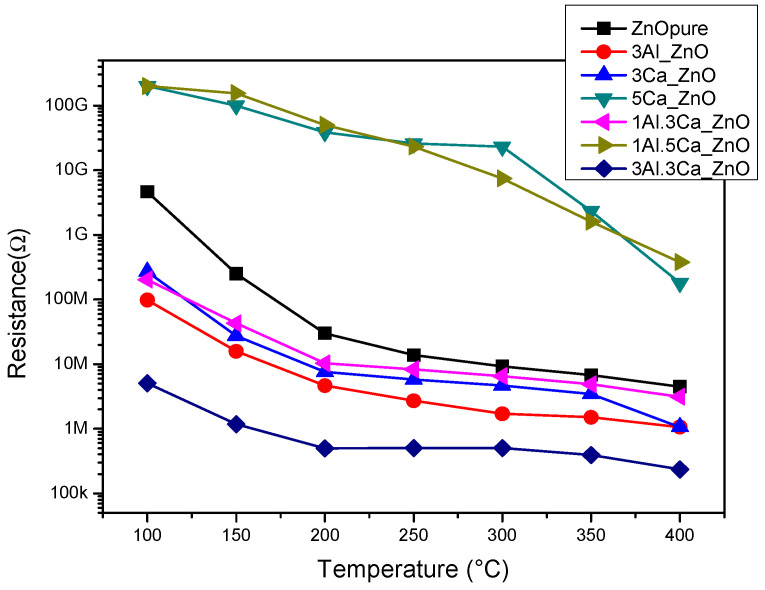
Baseline resistance in dry air of the pure and doped-ZnO sensors in the temperature range from 100 to 400 °C.

The baseline resistance in dry air of the Al,Ca doped/ZnO samples was first measured at different temperatures from 100 °C to 400 °C (see Figure 4). Pristine ZnO shows the typical behavior of metal oxide semiconductors, i.e., as the temperature increases the baseline resistance decreases, due to the increase in the charge carriers (electrons). 

The results of the investigation indicated also that the Al,Ca doped/ZnO samples behaved in a similar way, i.e., the introduction of the dopants does not change the intrinsic semiconductor characteristics of the pristine ZnO. However, as clearly observed in the graph, there is a significant decrease in the resistance of the ZnO due to the doping process. This could be due to the intrinsic defects that were induced during the doping process. The presence in the samples of the highest Ca loading (5 at%), led to a very high resistance (≥1 GΩ at temperature below 350 °C). These observations are in agreement with previous studies, highlighting that the electrical conductivity in the Al,Ca-doped ZnO system is dependent on the loading and the ratio between the two dopants [13,15].

**Figure 5:**
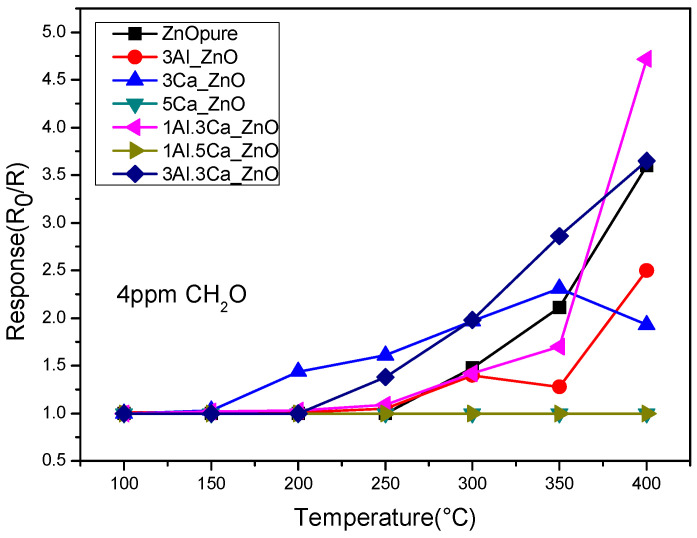
Responses to formaldehyde (4 ppm in dry air) of the pure and doped-ZnO sensors in the temperature range from 100 to 400 °C.

To investigate the sensing performance towards the detection of formaldehyde, the sensors were tested in the same temperature interval, from 100 °C to 400 °C with a target gas concentration of 4 ppm. Figure 5 shows that the response of the sensors towards formaldehyde increases with the increasing temperature, except for the sensors with the highest loading of Ca (i.e., 5 at%), which did not show any response to this gas. The effect of the Al/Ca ratio on the response to HCHO suggests that the presence of Al was effective in improving the response towards the target gas, compared to the corresponding Ca-ZnO sensors. A concentration of Al between 1 and 3% appeared to be most effective in the working temperature above 300 °C.

**Figure 6:**
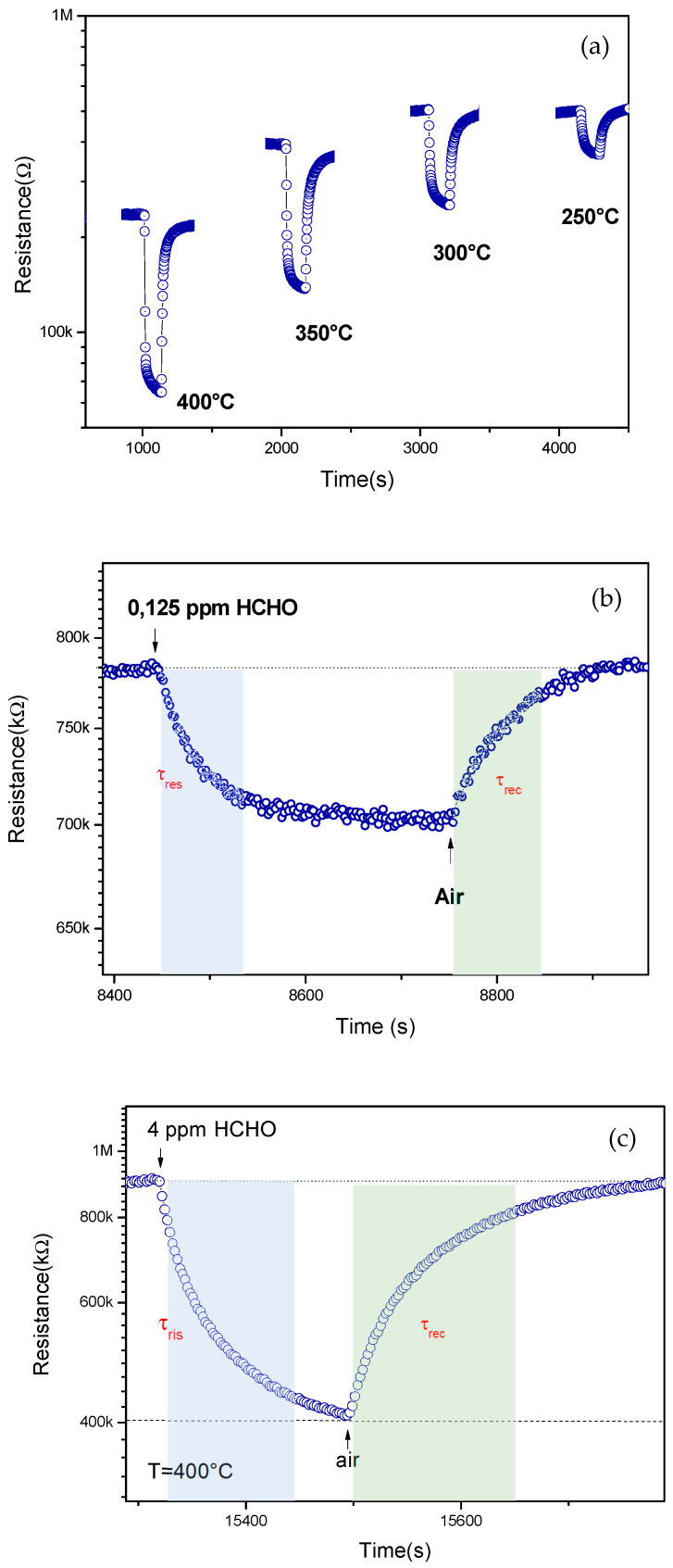
(**a**) Sensing tests of 3Al,3Ca-doped ZnO sensor to 4 ppm HCHO at different temperature; (**b**) Dynamic response of the sensor to 0.125 ppm HCHO at 400 °C, showing the response and recovery time; (**c**) Dynamic response of the sensor to 4 ppm HCHO.

Based on the electrical characterization and sensing tests at different temperatures, we selected the 3Al,3Ca doped ZnO sensor for further tests. This choice was motivated by the fact that this sensor showed a good balance of the sensing properties towards formaldehyde, e.g., a high response to the tested concentration of formaldehyde (4 ppm) and also a low baseline resistance. Figure 6a shows the dynamic response of this sensor towards 4 ppm HCHO at different temperatures. The highest response was found at the working temperature of 400 °C. Figure 6b,c shows the response of the sensor registered at this temperature when subjected to a pulse of 0.125 and 4 ppm of formaldehyde in dry air, respectively. It was found that the response (τ_res_), and also the recovery time (τ_rec_), registered at different concentrations, became longer with an increasing HCHO concentration (from 56 s at 0.125 ppm up to 100 s at 4 ppm for τ_res_ and from 60 s at 0.125 ppm up to 150 s at 4 ppm for τ_rec_). Following this, a transient HCHO pulse of 180 s duration and 300 s duration, respectively, for recovery in air was found.

**Figure 7:**
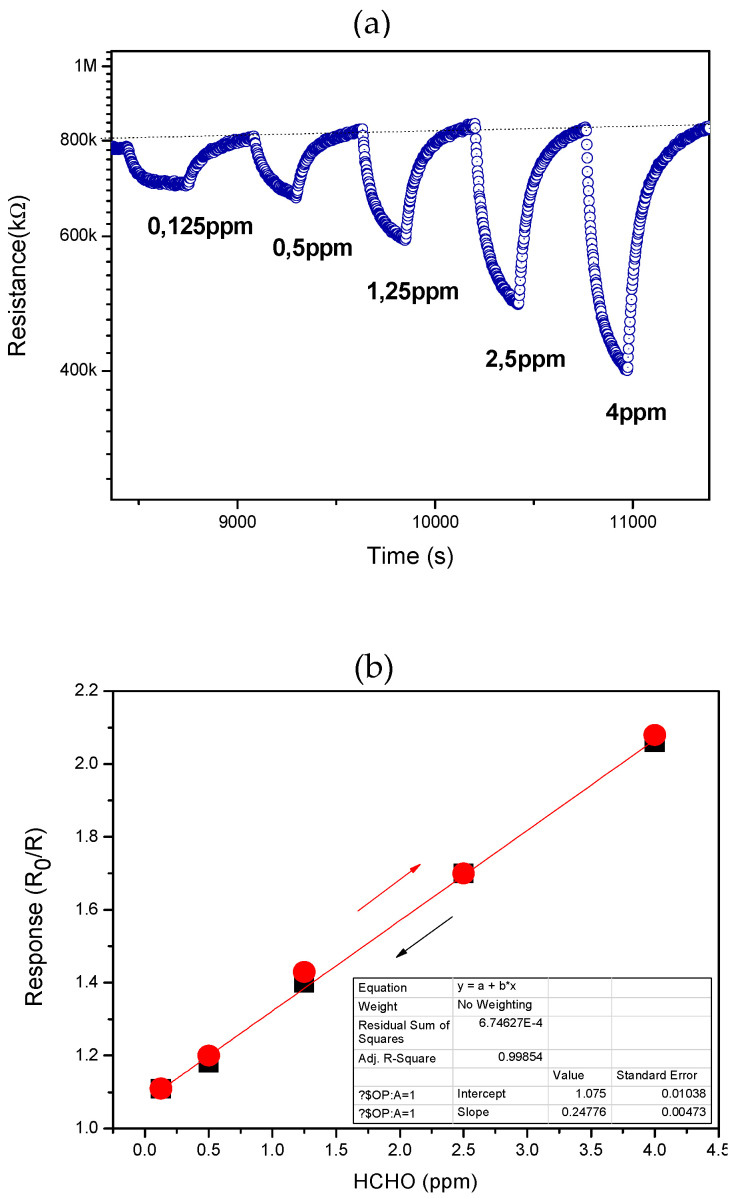
(**a**) Response of the 3Al,3Ca doped-ZnO sensor to different analyte concentrations, from 0.125 to 4 ppm; (**b**) Linear fit of response registered in the function of both increasing (red point) and decreasing (black point) HCHO concentration.

Figure 7a shows the response of the 3Al,3Ca-doped ZnO sensor at different concentrations of formaldehyde, increasing the values from 0.125 ppm to 4 ppm of HCHO. Even at the lowest concentration tested, the response signal satisfied the condition S/N > 3, where S is the response signal and N is the noise level, so the experimental limit of detection (LOD) was 125 ppb HCHO/air. The related calibration curve is reported in Figure 7b, which highlights the linearity of the response. The data have been fitted through a linear regression model (see the red line fitting the experimental data), resulting in a coefficient of determination R^2^ = 0.9985 which is indicative of the goodness of the linear fit. 

Furthermore, the response was registered by decreasing the concentration values of HCHO, from 4 ppm to 0.500 ppm. Data, plotted in the same graph of Figure 7b, demonstrate the absence of any hysteresis curve, i.e., practically the sensors do not suffer from the so called “memory effect”, which may invalidate the performance of the environmental sensors subjected to alternate, casual variation in the gas to be monitored. 

**Figure 8:**
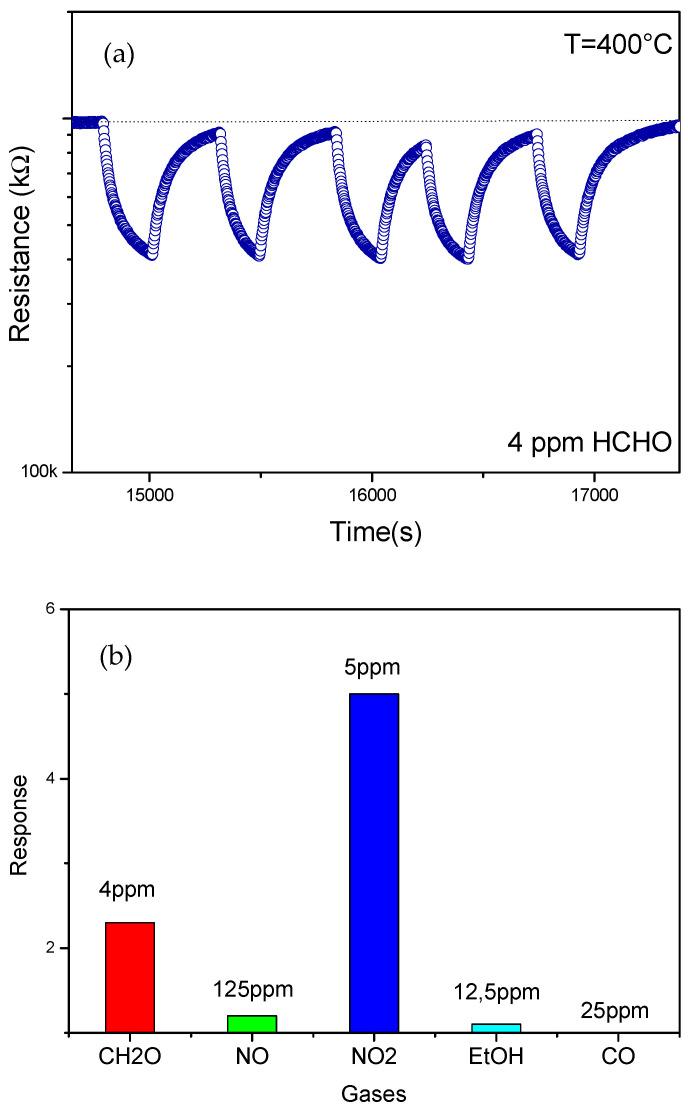
(**a**) Repeatability and reproducibility tests over time to 4 ppm HCHO at 400 °C; (**b**) Signal trend considering response and recovery time. Selectivity tests of 3Al,3Ca doped ZnO sensor to different gases.

To properly determine the sensor performance, the repeatability and reproducibility of the sensor are required. Repeatability is defined as the variance in the measured values of the target gas concentration upon repeated measurements. Reproducibility is the ability of the gas sensors to provide repeatability of the measurement results for prolonged usage. In Figure 8a the good repeatability and reproducibility of the signal is evident. The standard deviation determined from five replicates shows a low variability (<10%). 

Another parameter not to be overlooked is the selectivity of the sensor to formaldehyde. Figure 8b reports the response of the sensor to different indoor pollutants (i.e., NO, NO_2_, CO, ethanol) which can be found in indoor environments such as an office, an apartment, or a school environment. Among these gases, the sensor detects not only the formaldehyde but also the NO_2_. For NO_2_, an oxidizing gas, the baseline resistance variation is opposed (an increase of the resistance is found increasing the concentration) compared to HCHO, and this can be used to discriminate easily between the two gases. 

Sensitivity to other gases was not relevant, which indicated that the 3Al,3Ca doped ZnO sensor had good selectivity to formaldehyde. Selectivity is an important characteristic of the sensor because its functioning can be impaired from other gases present in the same ambient environment where the sensor is positioned. Here, we reported the response to some indoor gases at the concentrations we currently have in the laboratory. In particular, the possibility to monitor formaldehyde without interference from ethanol, is highly demanding for indoor applications [21]. Ethanol vapors are, for example, common in home environments such as the kitchen and this can cause false alarms. Usually, on metal oxide sensors, ethanol is a strong interferent, whereas the new optimized 3Al,3Ca doped ZnO sensor proposed here could overcome this limitation, being able to monitor HCHO with little or no interference from ethanol. However, we are aware that a more precise indication of the selectivity of the sensor can be acquired only by testing a larger number of different interferent gases. This is a priority of the study we plan to carry out in the future.

Finally, the sensing characteristics towards HCHO are compared with previous literature reports and presented in Table 1. This table shows some of the multitude of different materials used (single oxides, composites, and organic/metal oxide hybrids) and the related operating conditions (temperature and HCHO concentrations, etc.) in the HCHO conductometric sensors reported previously in the literature.

### 3.4. Sensing Mechanism and Final Considerations 

**Figure 9:**
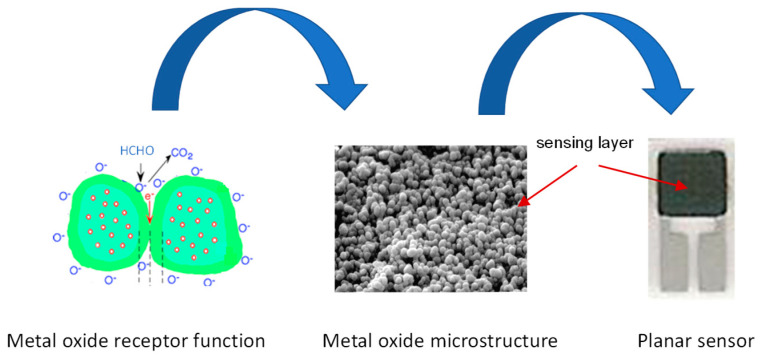
The functioning of an HCHO sensor detection.

A conductometric metal oxide gas sensor is based on the combined action of the receptor (the metal oxide sensing material) and an electrical transducer platform. The reception mechanism involves the interaction of the target gas on the surface of the metal oxide followed by the change of the sensing layer resistance (the transduction mechanism), as shown in Figure 9.

More precisely, the sensor exposed to air interacts with oxygen molecules that adsorb on the surface of the grains. In the case of a pure ZnO-based sensor, given that this metal oxide is a n-type semiconductor, the adsorbed oxygen ions extract electrons from the conduction band, generating a charge depletion layer which results in an increase in the potential barrier at grain level and an increase in sensor resistance. The depletion layer extension depends on the amount of adsorbed oxygen and the operating temperature of the sensor. In the presence of formaldehyde, a reducing gas, the interaction with an n-type semiconductor leads to decreases in sensor resistance due to the reduction of the bridge barrier.

The presence of the Al and Ca dopants in the ZnO determines several changes (morphological, microstructural, and electrical). The changes in electrical properties were due to the modification of Schottky and Frenkel defects in the structure of the ZnO crystal lattice, leading to a decrease/increase of the baseline resistance (see above in Figure 4). Based on their electrical characteristics, Ca ions were acting as electron attractors and Al ions as electron donors. The marked differences were also related to acid-base characteristics of the two dopants, with the Ca ions having a high capacity to increase the surface basicity of the composite, while Al ions increased the amount of acid sites. This means that the interaction of the HCHO with the sensing layer was dependent on the Al and Ca loading and their ratio. 

**Figure 10:**
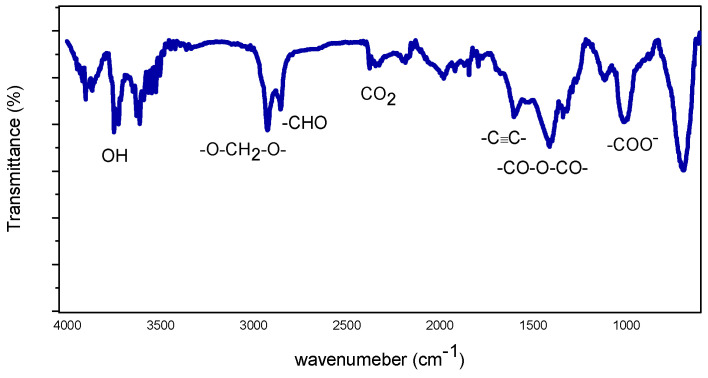
ATR-FTIR spectrum of 3Al,3Ca doped ZnO sensor after contact with HCHO.

For example, the strong interaction of the 3Al,3Ca doped ZnO sensor sensing layer with the formaldehyde was monitored by the ATR-FTIR spectrum (see Figure 10), highlighting the presence of prominent bands associated with the formaldehyde at 2925 cm^−^^1^ and 2855 cm^−^^1^ and attributed to the O-CH_2_-O- and the -CHO groups, respectively. 

These results concur with a computational study reporting the impact of Ca ions’ doping on the enhancement of formaldehyde adsorption, as demonstrated by the increase of HCHO adsorption energy on the surface from −4.2 to −36.1 kcal/mol [27]. These characteristics are the basis of the large sensing differences reported in the detection of formaldehyde with the various composite-based sensors investigated. Following on from the scope of the present paper, a deeper “in situ” AT-FTIR characterization of the composite materials under HCHO sensing in different working conditions could be of valuable interest for elucidating the HCHO sensing mechanism.

Focusing attention on practical application, the developed sensor has the advantage of a simple planar structure and can be easily fabricated for mass production by using low-cost materials and fabrication methods. Indeed, for the efficient production of gas sensors for the market, the manufacturing technology should be easy and inexpensive [28].

Here, both the base metal oxide (ZnO) and the dopants (Al, Ca) can be considered as appropriate for the low cost of the raw materials. Also, the synthesis procedure relies on a simple, easy, solution-processable wet-chemistry method. Fabrication of the final transducer device could include acceptable sensing layer printing processes such as screen printing or spray coating on a preformed interdigitated planar ceramic platform, ensuring further outstanding advantages such as advanced mechanical and electrical characteristics [29]. By considering these characteristics, the developed sensor would be most promising as a gas sensor for formaldehyde detection in indoor and workplace environments. 

## 4. Conclusions

In summary, the performances of a conductometric HCHO gas sensor based on the Al,Ca-doped zinc oxide composite have been presented. The results demonstrated that by optimizing the doping loading, a promising sensitive material showing good sensitivity, selectivity, and reproducibility for monitoring the low concentration of HCHO in air, was obtained. Thus, the developed 3Al,3Ca doped ZnO sensor could find applications in the indoor air and workplace pollution fields, due to additional advantages such as its easy fabrication and low cost.

## Figures and Tables

**Table 1:** Sensing characteristics of 3Al,3Ca doped ZnO sensor towards HCHO compared with previous literature reports.

Sensor	Temp. °C	HCHO Conc. (ppm)	Response (R_0_/R)	Ref.
**Au/In_2_O_3_**	100	50	85.7	[22]
**SWCNT@ZnO**	RT	500	1.2	[23]
**γ-Fe_2_O_3_**	320	100	6	[24]
**1.0 wt% MnO_2_-ZnO**	320	200	27	[25]
**Ga-doped ZnO**	400	205	13	[26]
***This work***	*400*	*4*	*3.5*	*-*
